# Object Naming After Thalamic Damage: Evidence From a Large-Scale, Chronic-Phase Study of Left Hemisphere Stroke Survivors

**DOI:** 10.1162/NOL.a.231

**Published:** 2026-03-19

**Authors:** Jie Zhang, Douglas Neville, Storm Anderson, Sophie M. Roberts, Thomas M. H. Hope, Alex P. Leff, David W. Green, Cathy J. Price

**Affiliations:** Center for Rehabilitation Medicine, Rehabilitation & Sports Medicine Research Institute of Zhejiang Province, Department of Rehabilitation Medicine, Zhejiang Provincial People’s Hospital, Affiliated People’s Hospital, Hangzhou Medical College, Hangzhou, China; Department of Imaging Neuroscience, Queen Square Institute of Neurology, University College London, London, UK; Department of Psychological and Social Sciences, John Cabot University, Rome, Italy; Institute of Cognitive Neuroscience, University College London, London, UK; Department of Brain Repair and Rehabilitation, University College London, London, UK; Department of Experimental Psychology, University College London, London, UK

**Keywords:** language recovery, lesion–symptom mapping, naming, stroke, thalamus

## Abstract

Functional imaging and clinical cases implicate the left thalamus in object naming, yet the prevalence of naming impairment after focal thalamic damage is low with variable impact and often rapid resolution. This suggests that compensatory mechanisms, within or beyond the thalamus, may support recovery. We hypothesized that thalamic damage would (a) not cause chronic anomia if other naming-related regions remain intact but (b) exacerbate anomia when co-occurring with damage to non-thalamic naming regions. To test these hypotheses, we retrospectively assessed naming ability in 550 left hemisphere chronic stroke survivors (52% with anomia). Lesion sites included focal thalamic lesions (*n* = 14), combined thalamic and non-thalamic lesions (*n* = 271), and lesions sparing the thalamus (*n* = 265). Whole-brain lesion–symptom mapping (LSM), using multivariate support vector regression, identified brain regions where damage was significantly related to naming ability. Contributions of different thalamic subregions to naming were assessed using ridge regression. Focal thalamic lesions were not associated with chronic anomia. LSM identified two naming-related clusters: a temporoparietal region of interest (ROI-TP) and a subcortical–insular region of interest (ROI-SC) including the lateral thalamus. However, lesion load in the lateral thalamus did not independently contribute to naming performance when controlling for damage to other parts of the ROI-SC, nor did any thalamic nuclei show additive effects beyond the ROI-TP and the non-thalamic ROI-SC. These findings suggest that thalamic damage in the dominant hemisphere does not cause long-term anomia in chronic stroke. Future research therefore needs longitudinal designs to track the trajectory of transient thalamic effects from the acute to chronic phases and to investigate whether naming impairments after thalamic lesions are (a) lesion specific but context dependent, emerging under increased cognitive load, or (b) attributable to non-lesion-site-dependent post-stroke factors such as fatigue.

## INTRODUCTION

The left thalamus has been implicated in the neural architecture subserving object naming, with converging evidence from neurosurgical stimulation (Ojemann, 1975), functional neuroimaging (Llano, 2013), and lesion studies of stroke patients (Crosson, 2013, 2021a; Hebb & Ojemann, 2013; Llano, 2013). Here, we review the existing evidence and outline the rationale and hypotheses for our investigation of object naming impairments (anomia) in a large cohort of stroke survivors, with and without thalamic damage, tested in the chronic post-stroke phase.

Functional neuroimaging in healthy individuals has revealed consistent bilateral thalamic activation with leftward dominance across various language paradigms, including naming, verbal fluency, lexical decision, reading, and tasks requiring working memory or complex perceptual processing (Llano, 2013). Other imaging evidence from the neurotypical population indicates thalamic involvement in executive functions (Bulut & Hagoort, 2024; Eickhoff et al., 2012; Yarkoni et al., 2011), including the control and regulation of multiple languages (Korenar et al., 2023; Wu et al., 2022). As successful naming has been related to the preservation of executive functions (Higby et al., 2019), thalamic activation during naming may reflect domain-general cognitive resources such as executive control or working memory, rather than core lexico-semantic retrieval processes alone. This proposal would explain why thalamic activation during object naming was observed relative to low-level baselines that did not control for perceptual processing, speech production, or executive function and failed to reach significance when these factors were controlled using high-level baselines (Price et al., 2005). This view aligns with emerging functional and neurophysiological evidence that the thalamus participates in reciprocal cortico-thalamo-cortical loops that regulate information flow rather than merely relaying sensory input (Crosson, 2021b).

A corticothalamic network-level perspective also underpins the interpretation of aphasic symptoms, such as anomia following thalamic stroke. Symptoms arise not solely from damage to classical language cortex per se but also from disrupted connectivity within broader cortico-subcortical networks that support language tasks such as naming (Rangus et al., 2024; Stockert et al., 2023).

Despite the consensus that the thalamus contributes to naming, the prevalence of anomia after thalamic stroke is consistently reported to be low across studies: 12% (6/52) in Fritsch et al. (2020), 19% (10/52) in Rangus et al. (2022), 12% (12/101) in Stockert et al. (2023), and 18% (15/85) in Rangus et al. (2024); however, only Fritsch et al. (2020) reported hemisphere-specific rates, with 18.75% (6/32) anomia in left-sided cases and 0% (0/20) anomia in right-sided cases. The severity of the naming impairment is also variable. For example, in Rangus et al. (2024), 4/15 patients were categorized with severe anomia; 4/15, with moderate anomia; and 7/15, with mild anomia. Many factors could contribute to low prevalence and variability after thalamic stroke. We start by considering two specific possibilities related to (a) the particular nucleus affected and (b) the timing of post-stroke naming assessment.

Lesions in multiple thalamic nuclei have been frequently implicated in case reports and small cohort studies of people with thalamic anomia, including the left anterior nucleus (Fritsch et al., 2020; Ghika-Schmid & Bogousslavsky, 2000; Mori et al., 1986; Nishio et al., 2011; Radanovic et al., 2003; Rangus et al., 2024), the mediodorsal nucleus (Stockert et al., 2023), the ventrolateral nucleus (Ojemann, 1975), and the pulvinar and lateral posterior nuclei (Radanovic & Almeida, 2021; Radanovic et al., 2003). A lack of consensus was also reported in a recent review that concluded there is no clear evidence linking patient differences in aphasic symptoms to specific lesion locations within the thalamus (Fritsch et al., 2022). Functional imaging evidence has also not yet clarified which thalamic nuclei contribute to naming.

For the second question, interindividual variability, the timing of post-stroke assessment appears to be critical. Anomia is most often identified in the acute or early subacute phase (Fritsch et al., 2020; Ghika-Schmid & Bogousslavsky, 2000; Kuljic-Obradovic, 2003; Rangus et al., 2022; Stockert et al., 2023), with reports that symptoms can resolve rapidly (Afzal & Farooq, 2013; Ghika-Schmid & Bogousslavsky, 2000; Kuljic-Obradovic, 2003; Ozeren et al., 1994; Tantik Pak et al., 2021). Rapid recovery alone, however, cannot fully explain the low prevalence of anomia even during the acute phase (Rangus et al., 2022, 2024; Stockert et al., 2023). Other factors must clearly be at play to explain interindividual variability in naming ability in patients with isolated thalamic lesions.

To frame the theory and motivation for the present study, we propose that inter-patient variability in naming outcomes may emerge from non-focal symptoms and/or lesion site–specific effects. Non-focal, stroke-related factors can transiently impair language function independently of the lesion location (Rohrer et al., 2008). For example, naming difficulties have been variably associated with fatigue (Mirman et al., 2024), hypertension (Albert et al., 2009), and depression (Georgieff et al., 1998). When these symptoms resolve, word-finding difficulty may improve. To attribute anomia to lesion site factors rather than generic stroke-related factors, one would ideally show that thalamic damage leads to higher prevalence and longer persistence of anomia compared to lesions elsewhere. To our knowledge, this question has not yet been addressed and lies beyond the scope of the current study, which pursues the alternative possibility that thalamic anomia is a direct consequence of thalamic damage.

To account for lesion-specific variability, including the often transient nature of naming deficits caused by thalamic lesions, we turn to the principles of redundancy and degeneracy (Price & Friston, 2002). Redundancy would be inferred if the thalamus houses more neurons and connections to language regions than are required for successful naming, allowing partial damage to be compensated for by surviving thalamic tissue. Degeneracy, by contrast, refers to the capacity of structurally distinct neural circuits to support the same function (Seghier & Price, 2018). These compensatory strategies or mechanisms might be engaged immediately to preserve function in the acute post-stroke phase and/or develop gradually through relearning and neuroplasticity, leading to symptom improvement over time. Critically, however, if all potential systems are compromised, functional recovery would not be possible.

Assuming that anomia can be caused by thalamic lesions, reports of recovery following such damage (Afzal & Farooq, 2013; Ghika-Schmid & Bogousslavsky, 2000; Kuljic-Obradovic, 2003; Ozeren et al., 1994; Tantik Pak et al., 2021) imply that the role of the thalamus in naming must have been either redundant or replaceable by other neural pathways (degeneracy). Complementary evidence also suggests that the thalamus may help support recovery from damage to non-thalamic regions. For example, functional magnetic resonance imaging (fMRI) activation in the undamaged thalamus correlated positively with improvements in naming performance post-stroke (Nenert et al., 2017).

Taken together, we hypothesize that anomia may be more likely to persist in individuals with damage to both thalamic and non-thalamic naming-related regions, compared to individuals with damage limited to one site. We test this hypothesis in a large retrospective cohort of left hemisphere stroke survivors in the chronic phase, by identifying lesion locations associated with anomia and examining whether concurrent damage to the thalamus and non-thalamic naming areas increased the likelihood or severity of anomia. In this study, rather than specifying the precise cognitive processing subserved by the thalamus during naming (e.g., lexico-semantic or executive), we focused on delineating which thalamic nuclei, if any, are reliably associated with chronic anomia in our sample.

## MATERIALS AND METHODS

### Study Participants

This was a retrospective study based on patient data selected from the Predicting Language Outcome and Recovery After Stroke (PLORAS) database (Seghier et al., 2016). Inclusion criteria were as follows: (a) unilateral stroke in the left hemisphere, visually inspected by an experienced neurologist using structural magnetic resonance imaging (MRI) images; (b) chronic stroke stage (≥6 months post-onset); (c) age at stroke onset >18 years; (d) native English speaker; and (e) right-handed pre-stroke. Patients were excluded if they had (a) any preexisting neurological or psychiatric history, (b) contraindications to MRI (e.g., claustrophobia), (c) incomplete language assessments, or (d) self-reported or clinician-observed visual impairment that would preclude reliable performance on object-naming tasks. A total of 550 patients met these criteria and were included in the current study. The study received ethical approval from the London Queen Square Research Ethics Committee in line with the Declaration of Helsinki. Written informed consent was obtained from all participants before enrollment.

### Neuropsychological Assessment

Language performance was assessed using the Comprehensive Aphasia Test (CAT), which evaluates six language domains: naming, repetition, comprehension (spoken and written), picture description, reading, and writing (Swinburn et al., 2004). Each domain score was converted into a standardized T-score, and an average score across these six domains was computed to indicate aphasia severity. For this study, naming ability, the core outcome measure, was assessed via the spoken object-naming subtest in the CAT (Swinburn et al., 2004). The task involves naming 24 pictures of common objects that vary in lexical frequency, imageability, length, and animacy. Each item is scored from 0 to 2 based on accuracy and promptness, with a maximum total score of 48. A T-score ≤61 on this subtest indicated impaired naming (i.e., anomia), as determined by the CAT user manual. Verbal fluency was assessed via three CAT tasks: (a) semantic word fluency, measured as the number of different animal names produced within 1 min; (b) letter (or phonemic) fluency, measured as the number of different words beginning with the letter “s” produced within a minute; and (c) spoken picture description, which indexes spontaneous and narrative speech production.

Based on lesion location, the 550 chronic stroke patients were categorized into three groups: those with focal thalamic lesions (*n* = 14), those with combined thalamic and non-thalamic lesions (*n* = 271), and those with lesions sparing the thalamus (*n* = 265). Across the full sample, 284 patients (51.6%) exhibited anomia. Median object naming T-scores and other clinical characteristics for each group are provided in Table 1.

**Table 1.** Demographic, clinical, and behavioral characteristics by group in the full sample (*n* = 550).

Variable	Focal thalamic lesions	Combined thalamic and non-thalamic lesions	Lesions sparing the thalamus
Sample size	14	271	265
Sex, *n* (%)			
Female	8 (57.1)	72 (26.6)	94 (35.5)
Male	6 (42.9)	199 (73.4)	171 (64.5)
Anomia prevalence, *n* (%)	0 (0)	199 (73.4)	85 (32.1)

***Mdn* (IQR)**			
Age in years	57.4 (21.4)	59.9 (17.7)	60.6 (19.9)
Education in years since age 14	0 (5.3)	1 (5)	2 (5)
Years post-stroke	2.3 (4)	4.4 (5.4)	2.2 (3.1)
Total lesion volume in cm^3^	0.9 (1.1)	110.1 (118.8)	11.9 (37.3)
Lesion load (%) in left thalamus	6.5 (9)	32 (42)	0 (0)
CAT T-scores			
Mean score of modalities	67.3 (3.8)	53.8 (11.3)	63.7 (9.5)
– *Total naming*	73 (7.3)	56 (14)	68 (12)
– *Spoken picture description*	66 (8.8)	52 (12)	63 (10)
– *Spoken comprehension*	63 (6.5)	55 (13)	61 (11)
– *Written comprehension*	64 (12.3)	54 (12)	63 (11)
– *Repetition*	62 (12.3)	52 (13)	59 (12)
– *Reading*	71 (5)	53 (15)	66 (14)
Fluency	72 (7)	55 (13)	68 (12)
Semantic memory	60 (0)	60 (9)	60 (0)
Object naming	66 (7)	56 (15)	66 (13)

*Note*. Total naming in the CAT combines scores from spoken object naming, action naming, and word fluency scores into a single composite score. IQR = interquartile range; CAT = Comprehensive Aphasia Test.

### MRI Data Acquisition and Preprocessing

All participants were scanned at University College London’s (UCL’s) Wellcome Centre for Human Neuroimaging or the Birkbeck–UCL Centre for Neuroimaging, using one of four Siemens MRI scanners: 3T Trio (including Prisma upgrade), 1.5T Sonata, 1.5T Avanto, and 3T Allegra. Each acquired structural T1-weighted images, with 176 sagittal slices, a matrix size of 256 × 244, and a final resolution of 1-mm^3^ isotropic voxels. The quality of the scans was optimized by using the MPRAGE (magnetization-prepared rapid acquisition with gradient echo) acquisition sequence to enhance contrast between different brain tissues and the identification of different structures and features and research protocols designed to reduce noise and artifacts.

**Figure 1.**
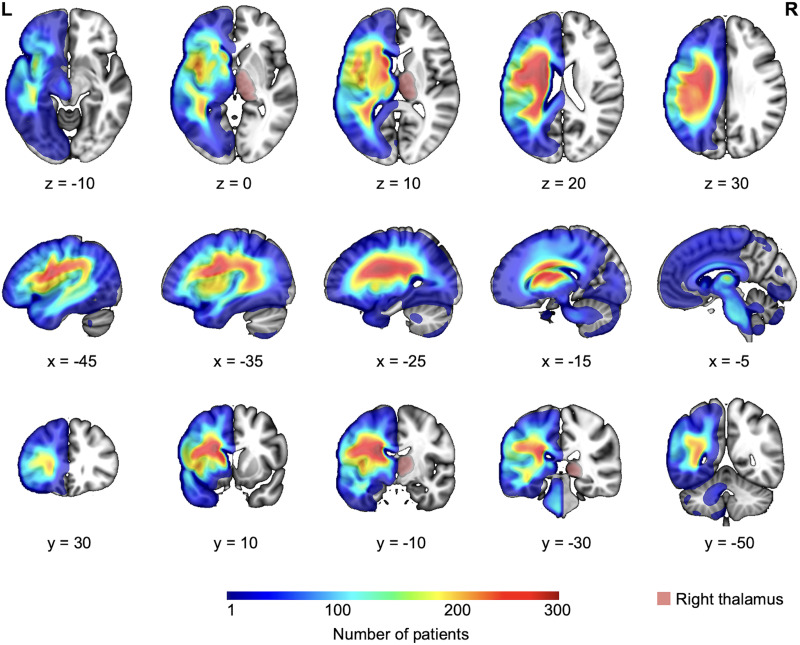
Overlap of stroke lesions from the total 550 patients. The contralateral (right) thalamus is highlighted in red.

**Figure 2.**
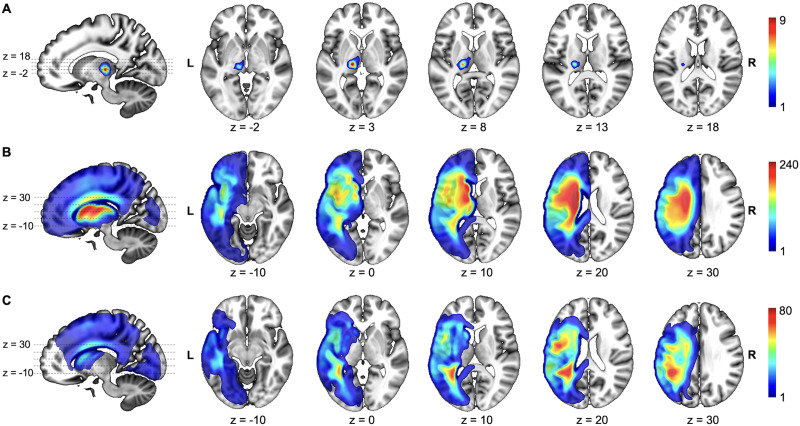
Overlap of stroke lesions by group. (A) Focal thalamic lesions (*n* = 14). (B) Combined thalamic and non-thalamic lesions (*n* = 271). (C) Lesions sparing the thalamus (*n* = 265). Note that the smallest thalamic lesion was 0.1 cm^3^ (= 100 mm^3^), which is 100 times larger than the 1-mm^3^ voxel resolution of the original scans.

Preprocessing was conducted in SPM12 (Department of Imaging Neuroscience, UCL; https://www.fil.ion.ucl.ac.uk/spm/) using MATLAB (The MathWorks, Inc.). To harmonize inter-scanner variability, raw images were transformed into quantitative probabilistic estimates of gray matter density. Images were then spatially normalized to MNI (Montreal Neurological Institute) space using a lesion-aware segmentation algorithm (Ashburner & Friston, 2005; Seghier et al., 2008) and smoothed with an 8-mm full width at half maximum Gaussian kernel, resulting in 2 × 2 × 2 mm voxels. Automated lesion detection was carried out on these smoothed, normalized images using a validated fuzzy clustering method, producing voxel-wise probability maps of abnormality relative to healthy controls scanned on the same scanner protocol used for the patients (Seghier et al., 2008). These fuzzy maps were binarized to create lesion masks using a threshold of 0.3, as recommended by Seghier et al. (2008), with a minimum lesion size of 0.8 cm^3^ (100 voxels), except for five patients with focal thalamic lesions that were smaller than 0.8 cm^3^. Each mask was reviewed, and if necessary, the threshold was adjusted to ensure alignment with expert neurological lesion description. A lesion overlap map for the whole sample is shown in Figure 1. Lesion overlap maps are shown for the whole sample (Figure 1) and for each subgroup: focal thalamic lesions (Figure 2A), lesions affecting both thalamic and non-thalamic regions (Figure 2B), and lesions sparing the thalamus (Figure 2C).

### Long-Term Language Outcomes Associated With Focal Thalamic Strokes

Focal thalamic strokes were identified by selecting all patients, within the full sample of 550, whose binary lesion masks showed damage confined to the left thalamus. Each lesion image was independently verified by an experienced neurologist. In addition, thalamic nuclei were defined using the Automated Anatomical Labeling atlas 3 (AAL3; Rolls et al., 2020), and lesion load was quantified as the proportion of voxels within each nucleus overlapped by the lesion mask. For each patient, demographic data, lesion load in each thalamic subregion, and CAT T-scores across all language domains were reported.

### Whole-Brain Analysis: Identifying Thalamic and Non-Thalamic Lesion Sites Associated With Anomia

To identify regions of interest (ROIs) where lesion load correlated with naming performance across the whole brain, multivariate lesion–symptom mapping (LSM) was implemented, entering data from the full sample into support vector regression LSM (SVR-LSM; Zhang et al., 2014). This machine learning approach models the complex relationships between distributed lesion patterns and behavioral outcomes by treating lesion patterns as high-dimensional predictors to learn which *particular combinations* of damaged voxels predict worse outcomes, accounting for inter-voxel dependencies and lesion volume confounds. Lesion maps were used as input features, with object naming T-scores as the dependent variable. The analysis was implemented in MATLAB, using SVR (gamma = 5, cost = 30), fivefold cross-validation, and permutation testing (*n* = 10,000) to assess statistical significance (voxel-wise *p* < 0.005, cluster-wise *p* < 0.05).

The whole-brain SVR-LSM was performed iteratively, with each iteration removing patients who had >10% lesion load in naming-related regions identified in the previous iteration (Gajardo-Vidal et al., 2018). This process continued until no additional significant clusters were identified. The rationale for this iterative LSM approach is to reduce false-negative results that can arise when a symptom, such as anomia, can result from lesions to multiple, spatially distributed regions within a neural system. For example, anomia might be observed in some patients who have damage to Region A but not Region B (A-not-B), whereas others show the opposite pattern (lesions in Region B but not Region A [B-not-A]). When both types are combined in a whole-brain LSM analysis, the lesion–symptom association at each region is weakened because, at the same voxel, some anomic patients have damage while others do not. This variability obscures true effects, leading to either null results (if group sizes are balanced, e.g., there are equal numbers of patients with damage to A-not-B and B-not-A) or the partial detection of the true distributed network (if one region is overrepresented, e.g., there are unequal numbers of patients with damage to A-not-B and B-not-A). Iteratively removing patients with damage to previously identified regions (e.g., Region A) reduces this heterogeneity, increases the signal-to-noise ratio, and improves detection of additional relevant lesion sites (e.g., Region B), until sample size becomes too small to support statistical inference.

The >10% lesion threshold was selected to balance two competing priorities: (a) excluding patients whose anomia could be attributed to previously identified regions, thereby increasing sensitivity for detecting new associations, and (b) preserving statistical power by retaining a sufficient sample size. We also tested >5% and >20% thresholds to assess robustness: A 5% threshold is more conservative, excluding more patients with partial overlap, whereas a 20% threshold maximizes power by retaining more patients (i.e., those with up to 19% lesion load).

### The Frequency of Anomia Following Damage to Different Combinations of ROIs

ROIs were defined based on the whole-brain iterative SVR-LSM analysis described above. To examine how different combinations of ROI damage related to anomia, we assigned each of the 550 patients to subgroups depending on whether specific ROIs were damaged or spared. We then calculated the frequency of anomia in patients with damage to (a) each ROI alone, (b) different combinations of ROIs, and (c) none of the ROIs. Group differences in anomia frequency across these groups were computed using odds ratios (*OR*s) of effect sizes with chi-square tests assessing their statistical significance.

Damage was defined as >5% lesion load to an ROI; and sparing, as <5%. This threshold was chosen because it ensured that ROIs labeled “undamaged” were at least 95% spared, minimizing misclassification due to partial damage. This conservative threshold for defining spared tissue makes the corresponding threshold for damaged tissue relatively lenient (any patient with at least 5% damage). However, we investigate the relationship between the degree of damage to the thalamus and naming performance in the subsequent analysis.

### Testing Whether the Thalamic Part of an ROI Contributes to Anomia

Our focus was on ROIs with damage to the thalamus. To determine whether lesion load specifically to the thalamic part of an ROI contributed to anomia, we subdivided each ROI into distinct atlas-defined subregions, including the thalamic parts. This subdivision was guided by the AAL3 atlas for gray matter tracts (Rolls et al., 2020) and by the human cross-species tractography (XTRACT) atlas for white matter tracts (Warrington et al., 2020).

To examine the relationship between naming performance and lesion load in each subregion, while accounting for the effects of lesion load in other subregions, ridge regression was chosen to address potential multicollinearity arising from the spatial proximity and interdependence of lesion load across subregions. In other words, when lesion load in one subregion correlates with lesion load in another, the lesion–symptom association is weakened. Ridge regression analyses were conducted in R software (Version 4.3.2; R Foundation for Statistical Computing). All regression models controlled for confounding variables, including age, sex, years of education, total left hemisphere lesion volume, and time post-stroke duration.

To investigate the interdependence of lesion load across subregions, we conducted post hoc analyses to assess how lesion load in the thalamic subregion correlated with lesion load in non-thalamic subregions, controlling for lesion size. We then tested whether combined damage to thalamic and non-thalamic parts of the ROI was more strongly associated with anomia than damage to each area alone, by comparing anomia frequencies across lesion-defined subgroups using *OR*s and chi-square tests. This involved analyzing the frequency of anomia for different combinations of subregional damage, using the approach described in the previous section, where we compared anomia rates related to damage in a single or multiple ROIs. The key difference being that we now focus on anomia rates across different patterns of damage within a single ROI.

### Comparing the Contribution of Different Thalamic Nuclei to Anomia

Here, we compared the effect of damage to six different thalamic nuclei on naming performance. As above, the thalamus was subdivided into anterior, lateral, intralaminar, medial, geniculate, and pulvinar nuclei. Lesion load in each of these areas was entered into a ridge regression, factoring out the influence of confounders, including total lesion volume, time post-stroke, and demographics. In follow-up analyses, we also factored out the influence of damage to the ROIs associated with naming in the SVR-LSM. All *p* values reported in this study are two-tailed.

## RESULTS

### Long-Term Language Outcomes Associated With Focal Thalamic Strokes

Out of the total 550 eligible patients with left hemisphere strokes in the chronic phase, only 14 (2.5%) presented with focal thalamic damage, with median time post-stroke of 2.35 years (interquartile range [IQR] 3.9) (Table 2). None had anomia or aphasic scores on any of the CAT verbal (spoken picture description, semantic word fluency, and letter fluency), comprehension, reading, or semantic memory subtests, but three had very mild difficulties with auditory repetition (Table 3). The thalamic subregions damaged in this sample are shown in Table 2, along with the demographic details for each patient. Among the 14 patients, the number of individuals with more than 10% damage to each thalamic nucleus was as follows: intralaminar (*n* = 8), pulvinar (*n* = 7), lateral (*n* = 4), medial (*n* = 3), anterior (*n* = 1), and geniculate (*n* = 1). See Table 2 for the exact lesion load in each region.

**Table 2.** Thalamic damage and demographic details for the 14 patients with focal thalamic stroke.

Patient ID	Age in years	Sex	Education (years after age 14)	Years post-stroke	Total lesion volume (cm^3^)	Lesion load in thalamic subregions (%)					
						Pulvinar	Anterior	Geniculate	Intralaminar	Lateral	Medial
PS0018	76.8	M	2	3.7	1.9	20	0	4	34	19	0
PS0587	37.2	F	0	0.9	0.9	23	0	0	0	1	0
PS0692	59.1	M	0	2.4	1.5	20	0	4	48	15	1
PS0013	59.8	M	0	1.5	1.0	17	0	0	20	9	1
PS0697	59.7	F	5	1.0	0.5	10	0	0	6	6	0
PS2754	55.3	F	0	1.1	3.0	38	0	2	52	17	73
PS2870	55.6	M	6	0.9	0.9	12	0	16	4	6	0
PS0059	82.0	F	0	6.4	0.1	0	0	0	8	2	0
PS0408	50.3	F	0	5.0	0.6	0	28	0	8	5	18
PS0411	45.4	M	0	5.0	0.2	0	0	0	20	2	0
PS0598	33.3	F	4	5.5	0.1	0	0	0	2	2	2
PS1479	52.6	F	11	3.6	0.9	8	0	0	18	8	0
PS1958	70.0	M	0	1.1	0.6	0	0	0	24	3	24
PS3116	71.9	F	8	2.3	1.6	7	0	0	66	19	1

*Note*. The top 7 rows are data on patients with ≥10% in the pulvinar. M = male; F = female.

**Table 3.** Comprehensive Aphasia Test scores in 14 patients with focal thalamic strokes.

Patient ID	Spoken object naming	Spoken picture description	Spoken comprehension	Total repetition	Fluency	Reading	Semantic memory
PS0018	66	60	74	59^a^	63	71	60
PS0587	74	73	62	59^a^	72	71	60
PS0692	66	72	60	58^a^	75	66	60
PS0013	64	66	63	62	68	66	60
PS0697	74	75	63	66	72	66	60
PS2754	62	62	62	61	60	71	60
PS2870	62	73	74	60	75	71	51
PS0059	66	61	60	66	68	71	51
PS0408	74	66	74	72	74	71	60
PS0411	66	64	67	72	75	71	60
PS0598	70	64	59	72	63	63	60
PS1479	74	74	67	62	75	66	60
PS1958	66	69	60	72	75	71	60
PS3116	70	66	67	60	69	71	60

*Note*. The top 7 rows are data on patients with ≥10% in the pulvinar.

^a^ Scores are within the aphasic range.

### Whole-Brain Analysis: Identifying Thalamic and Non-Thalamic Lesion Sites Associated With Anomia

In the first whole-brain LSM analyses (LSM-1) of data from the full sample of 550 left hemisphere stroke patients (284 with anomia), naming ability was significantly related to damage in a left temporoparietal region (temporoparietal region of interest [ROI-TP]), which included the superior and medial temporal cortex, inferior fronto-occipital fasciculus, middle longitudinal fasciculus, arcuate fasciculus, and inferior longitudinal fasciculus (Figure 2A).

**Figure 3.**
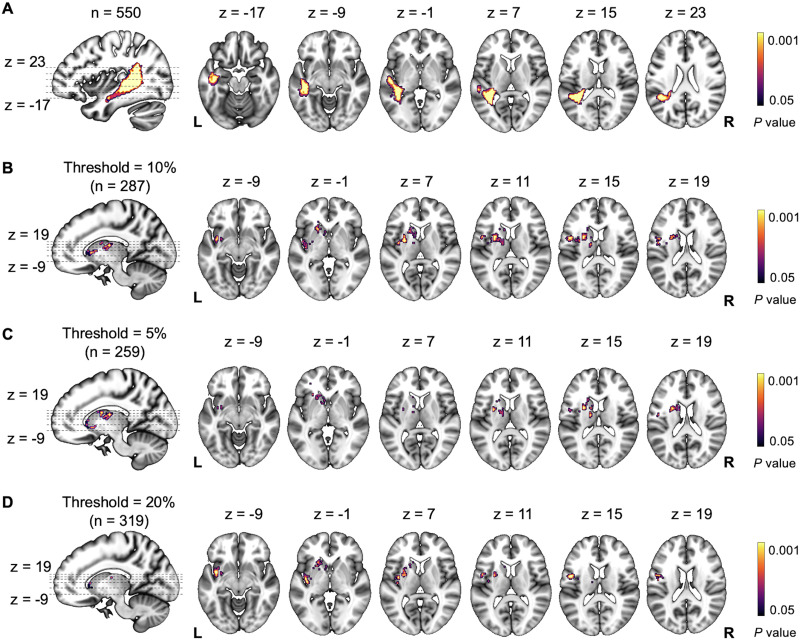
Lesion–symptom mapping (LSM) results for multivariate support vector regression. (A) Results from the first whole-brain LSM analyses, including the full sample (*n* = 550). (B–D) Results from the second whole-brain LSM analyses after excluding patients with temporoparietal region-of-interest damage according to the following lesion load thresholds: (B) 10% (*n* = 287), (C) 5% (*n* = 259), and (D) 20% (*n* = 319). All *p* values are two-tailed (voxel-wise *p* < 0.005, cluster-wise *p* < 0.05).

In the second whole-brain LSM analyses (LSM-2), we excluded patients with at least 10% lesion load to the ROI-TP and repeated LSM, using data from the remaining 287 patients (75 with anomia). This analysis detected a relationship between naming ability and damage to a subcortical-centered region (subcortical–insular region of interest [ROI-SC]) that included seven subregions (based on gray and white matter atlases): lateral thalamus, putamen, caudate, anterior thalamic radiation, superior thalamic radiation, and superior longitudinal fasciculus III (SLF-III), with cortical extension into the insula (Figure 3B). A similar ROI-SC cluster was observed when LSM-2 was repeated using a 5% lesion load threshold in the ROI-TP (Figure 3C), i.e., excluding more patients with a small amount of damage in the ROI-TP. However, the ROI-SC included little or none of the lateral thalamus when a 20% lesion load threshold was applied (Figure 3D), reflecting the inclusion of more patients with low lesion load in the ROI-TP, which may reduce sensitivity to other regions.

In the third whole-brain LSM analyses (LSM-3), we excluded patients with suprathreshold damage (>10%) to the ROI-TP (LSM-1), the ROI-SC (LSM-2), or both and repeated LSM on data from 180 patients (25 with anomia). In this analysis, no further lesion sites were significantly related to naming ability (*p* > 0.05 corrected; *p* > 0.001 uncorrected), even when varying the threshold for “damage” above (20%) and below (5%) the 10% threshold used in the previous analyses.

Across these analyses, two different regions were associated with naming ability: ROI-TP and ROI-SC. The only thalamic region associated with naming in the whole-brain analysis was the left lateral thalamus, which was part of the ROI-SC.

### The Frequency of Anomia Following Damage to Each, Both, or Neither ROI

**Figure 4.**
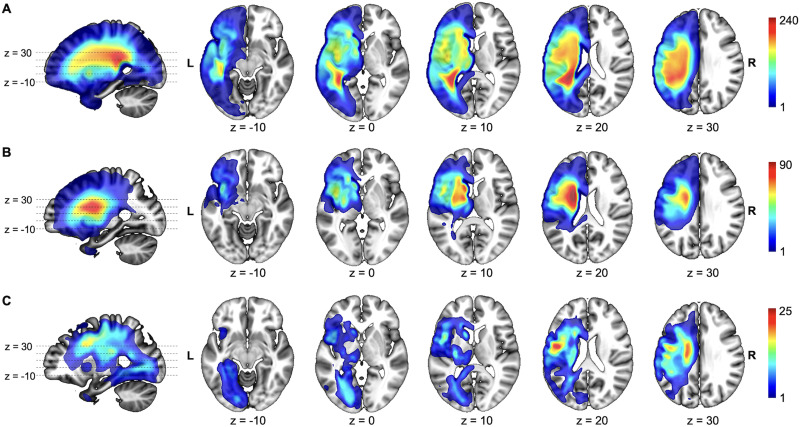
Overlap of stroke lesions by region-of-interest subgroup. (A) Temporoparietal region of interest (ROI-TP) group: patients with ≥10% lesion load in the ROI-TP (*n* = 263). (B) Subcortical–insular region of interest (ROI-SC) group: patients with <10% lesion load in the ROI-TP and ≥10% lesion load in the ROI-SC (*n* = 107). (C) Neither group: patients with <10% lesion load in both the ROI-TP and the ROI-SC (*n* = 180).

The ROI-TP and the ROI-SC were identified by regressing lesion load on naming scores. To calculate the frequency of anomia following damage to each ROI alone, both ROIs, or neither ROI, we split all 550 patients into four groups, defining “spared” as less than 5% lesion load to an ROI and “damaged” as 5% or more damage. Overlap of stroke lesions by ROI subgroup is shown in Figure 4. The frequency of anomia was highest (180/230 = 78%) when both ROIs were damaged and lowest (15/141 = 11%) when both ROIs were spared, with 66% (40/61) having anomia after damage to the ROI-TP but not the ROI-SC and 42% (49/118) having anomia after damage to the ROI-SC but not the ROI-TP. These results confirm that the frequency of anomia was significantly higher in patients with damage to either the ROI-TP or the ROI-SC compared to no damage to either region (ROI-TP: *OR* = 16.0, *χ*^2^ = 64.9; ROI-SC: *OR* = 6.0, *χ*^2^ = 32.9; both Bonferroni-corrected *p* < 0.001). Moreover, concurrent damage to both ROIs was associated with a significantly greater risk of anomia than damage to only one ROI (*OR* = 3.6, *χ*^2^ = 36.4; Bonferroni-corrected *p* < 0.001). Finally, damage to the ROI-TP alone was also significantly more likely to result in anomia than damage to the ROI-SC alone (*OR* = 2.7, *χ*^2^ = 9.3; Bonferroni-corrected *p* = 0.018).

### Which Parts of the ROI-SC Contribute to Anomia?

**Figure 5.**
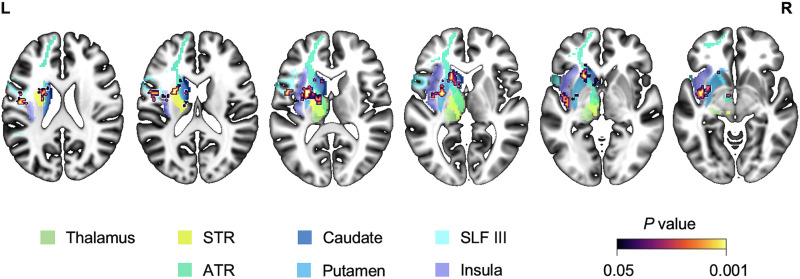
Anatomical definition of the subcortical–insular region of interest (ROI-SC) and its subdivisions. The composite ROI-SC mask included seven anatomically distinct regions: (a) thalamus, (b) superior thalamic radiation (STR), (c) anterior thalamic radiation (ATR), (d) caudate, (e) putamen, (f) superior longitudinal fasciculus III (SLF-III), and (g) insular cortex. These subregions were segregated using standard neuroanatomical atlases in MNI (Montreal Neurological Institute) space and are displayed here on axial slices.

To identify which of the seven parts of the ROI-SC were most strongly associated with naming ability, we calculated lesion load in each part for each of the 118 patients who had less than 5% damage to the ROI-TP (thereby excluding the influence of ROI-TP damage on naming scores). A visualization of the anatomical segmentation of the ROI-SC is provided in Figure 5, illustrating the seven subregions defined by standard atlases. Lesion load was regressed with naming scores, using ridge regression to control for multicollinearity in the degree of damage to different parts. The analysis identified significant associations between naming ability and lesion load in only two of the seven parts (Table 4): SLF-III (standardized *β* = −41.679, 95% CI [−56.815, −26.543], *p* < 0.001) and the caudate (standardized *β* = −24.343, 95% CI [−45.181, −3.505], *p* = 0.023).

**Table 4.** Ridge regression analysis of object naming scores based on lesion load in the subcortical–insular region of interest (ROI-SC) subregions (at 5% lesion load thresholds, *n* = 259).

Variable	Standardized *β* (*SE*)	95% CI	*t*	*p* (2-tailed)
Age	−16.511 (5.81)	[−27.898, −5.124]	−2.842	0.005**
Sex at birth	6.738 (5.802)	[−4.633, 18.110]	1.161	0.2466
Education	0.923 (5.667)	[−10.185, 12.031]	0.163	0.8708
Time post-stroke	32.119 (5.803)	[20.746, 43.492]	5.535	<0.001***
Total lesion volume	6.982 (8.956)	[−10.571, 24.535]	0.780	0.4364

Lesion load in ROI-SC subregions				
– *Lateral thalamus*	−3.883 (11.009)	[−25.459, 17.694]	−0.353	0.7246
– *Insula*	−13.044 (8.033)	[−28.789, 2.700]	−1.624	0.1057
– *Caudate*	−24.343 (10.632)	[−45.181, −3.505]	−2.290	0.023*
– *Putamen*	−9.067 (11.164)	[−30.948, 12.814]	−0.812	0.4175
– *ATR*	−12.990 (10.422)	[−33.418, 7.438]	−1.246	0.2138
– *STR*	7.939 (11.603)	[−14.803, 30.680]	0.684	0.4945
– *SLF-III*	−41.679 (7.723)	[−56.815, −26.543]	−5.397	<0.001***

*Note*. CI = confidence interval; ATR = anterior thalamic radiation; STR = superior thalamic radiation; SLF-III = superior longitudinal fasciculus III. *p < 0.05, **p < 0.01, ***p < 0.001.

The absence of significant relationships between anomia and lesion load in other parts of the ROI-SC does not imply a lack of contribution to naming score. Rather, effects in these regions may have been smaller or shared with the regions that showed stronger associations. For example, 69% (51/74) of patients with >5% damage to the SLF-III part also had >5% damage to the insula part, and the frequency of anomia was significantly higher (*OR* = 4.59, *χ*^2^ = 11.8; Bonferroni-corrected *p* < 0.001) when SLF-III and the insula were both damaged (32/51 = 63%) than when SLF-III was damaged sparing the insula (6/23 = 26%) or the insula was damaged sparing SLF-III (5/18 = 28%), with no significant difference between SLF-III-only damage versus insula-only damage (26% versus 28%: *OR* = 0.92, *χ*^2^ = 0.0; Bonferroni-corrected *p* > 0.05). In other words, the combination of damage to SLF-III and the insula was most critical to naming.

Post hoc analyses were conducted to determine whether damage to the lateral thalamus was causally related to anomia or incidental to it. This involved examining the frequency of anomia across subgroups with different lesion combinations. We note that all 77 patients with >5% damage to the caudate part of the ROI-SC also had >5% damage to the lateral thalamic part, and there were no patients with damage to the lateral thalamic part sparing the caudate (or vice versa). This pattern indicates that the caudate and lateral thalamic parts were invariably co-damaged in our cohort, preventing us from dissociating their individual contributions and permitting assessment of their combined effect only. However, this limitation proved inconsequential because the incidence of anomia was not significantly different in patients with damage to both the lateral thalamus and caudate and those in which both regions were spared (*p* > 0.05). Moreover, this pattern held across four subgroup comparisons: patients with (a) damage to both SLF-III and the insula (26/40 = 65% vs. 6/11 = 55%), (b) damage to SLF-III but not the insula (1/5 = 20% vs. 5/18 = 28%), (c) damage to the insula but not SLF-III (5/15 = 33% vs. 0/0), and (d) sparing of both SLF-III and the insula (5/17 = 29% vs. 1/9 = 11%).

In summary, in this section, we found no significant evidence that (a) naming score was related to lesion load in the left thalamus (regression analysis) or (b) the frequency of anomia was higher when the lateral thalamus was damaged versus spared.

### Comparing the Contribution of Different Thalamic Nuclei to Anomia

When considering the full sample of 550 patients, lesion load across the whole thalamus was significantly related to object naming scores after factoring out the influence of total lesion volume, age, and time post-stroke (standardized *β* = −32.35, 95% CI [−57.23, −7.47], *p* = 0.011). When the analysis was repeated replacing lesion load across the whole thalamus with lesion load estimates for the six distinct thalamic subregions, only lesion load in the pulvinar subregion was significantly related to naming ability (standardized *β* = −36.38, 95% CI −67.62 to −5.13, *p* = 0.023), with no significant effect in any of the other five thalamic subregions (*p* > 0.05). However, when the analyses included additional regressors to account for lesion load in non-thalamic regions associated with naming in the whole-brain analysis (i.e., ROI-TP, insula, and SLF-III), the significant effect in the pulvinar subregion, or the whole thalamus, was no longer significant (*p* > 0.05).

Additionally, we found no significant relationship between object naming scores and the degree of damage to any thalamic region (*p* > 0.05) irrespective of whether these ridge regression analyses were restricted to subsets of patients with damage to the ROI-TP and the ROI-SC (*n* = 188), the ROI-TP but not the ROI-SC (*n* = 75), the ROI-SC but not the ROI-TP (*n* = 107), or neither ROI (*n* = 180). None of these findings support our hypothesis that naming scores would be lower when thalamic damage co-occurred with damage to other naming regions.

## DISCUSSION

Prior literature has shown that approximately 10%–20% of patients with focal thalamic lesions present with anomia during the acute post-stroke stage, with most cases recovering rapidly over time. We therefore hypothesized that patients with focal thalamic lesions would rarely, if ever, have anomia that persisted months or years post-stroke. Any observations of anomia in these patients, however, were of interest for considering the many possible reasons for inter-patient variability, including (a) differential involvement of specific thalamic nuclei affected; (b) variability in the timing of post-stroke assessment; (c) functional redundancy within the thalamus, whereby spared regions can compensate for partially damaged areas; (d) neural degeneracy, in which alternate neural circuits support naming after thalamic damage; and (e) generic stroke-related factors, such as physiological or psychological stress, that are independent of the anatomical locus of the lesion.

Based on models of distributed language networks, we also hypothesized that multiple neural networks can independently support naming, with the thalamus serving as a key node in one (or more) of these networks. Under this framework, anomia should be more frequent or severe when both the thalamic and non-thalamic regions are damaged, compared to when the non-thalamic network is damaged but the thalamus is spared. The rationale was that multisite damage could limit neuroplasticity, disrupt interregional coordination, or impair executive functions such as attention and working memory, all of which are necessary for naming.

In our large-scale, chronic-phase study of left hemisphere stroke survivors, we found no evidence supporting either hypothesis. Thalamic lesions neither caused nor exacerbated long-term anomia. This outcome was observed both in (a) 14 patients with isolated thalamic damage and (b) the analyses examining whether thalamic damage exacerbated naming deficits when co-occurring with lesions to other naming-related regions. Below, we consider these null findings and discuss their implications for understanding thalamic function and post-stroke language recovery.

### The Absence of Long-Term Anomia Following Focal Thalamic Lesions

The absence of anomia in our 14 patients with chronic, focal thalamic lesions indicates that object naming can be sustained even when left thalamic connections are disrupted. This is consistent with earlier reports showing that acute-stage thalamic anomia is typically transient (Ghika-Schmid & Bogousslavsky, 2000; Kuljic-Obradovic, 2003). Several factors may account for the preserved naming abilities observed in the context of small focal thalamic lesions.

First, not all thalamic nuclei may be necessary for naming. For example, although anterior thalamic damage was observed in all six of the patients with acute anomia in Fritsch et al. (2020), only one patient in our chronic sample of 14 patients with focal thalamic lesions had >10% anterior thalamic damage. In contrast, >10% damage was more frequently observed in the intralaminar (*n* = 8), pulvinar (*n* = 7), lateral (*n* = 4), medial (*n* = 3), and geniculate (*n* = 1) nuclei, with most patients exhibiting partial damage across multiple nuclei (Table 2). Second, individual nuclei were only partially rather than fully damaged, potentially sparing functional tissue and connections that support naming, consistent with the concept of redundancy. Future studies could address this possibility by testing long-term naming impairments in a larger cohort of patients with a greater sampling of damage to each thalamic subregion. Third, the damaged thalamic regions may have contributed to naming performance pre-stroke, with regions outside the thalamus compensating for focal thalamic damage post-stroke, by engaging an alternative neural network for naming—in line with the concept of neural degeneracy. This hypothesis could be tested in future work via fMRI, showing either upregulation of non-thalamic activation after thalamic lesions or additive effects of thalamic and extra-thalamic damage on naming. The fourth explanation is that the naming impairments observed after focal thalamic lesions in the acute stage may reflect stroke-related factors, such as fatigue (Mirman et al., 2024), elevated blood pressure (Albert et al., 2009), or mood disturbances like depression (Georgieff et al., 1998), rather than lesion-specific disruption of language systems. To clarify the specificity of thalamic contributions rather than these generic factors, future studies could test whether anomia is more frequent and enduring after focal thalamic lesions compared to strokes affecting other regions.

In addition, several focal thalamic lesions in our sample were very small (<1 cm^3^). Although all anatomical images were acquired at 1-mm isotropic resolution, subsequent normalization and smoothing may have increased partial-volume effects, limiting the precision with which damage could be assigned to individual subnuclei. This methodological limitation reduces confidence in subnuclear localization, particularly in the smaller lesions, and reinforces the need for future work with higher resolution imaging to address questions at the fine-grained subnuclear level.

Moreover, the lesion distribution patterns across subgroups are consistent with known vascular territories. Isolated thalamic infarcts reflect small-vessel strokes within the posterior circulation, whereas combined thalamic and non-thalamic lesions predominantly involved the middle cerebral artery (MCA) territory, often affecting the perisylvian cortex along with underlying white matter and subcortical nuclei. Lesions sparing the thalamus also clustered within the MCA territory but with a slightly more posterior distribution. These vascular differences help explain why the additive impact of damage to MCA-supplied perisylvian regions disrupted naming ability in the long term. Nevertheless, because our study did not systematically classify strokes by vascular territory, future work combining lesion mapping with angiographic or perfusion data could further clarify how anterior–posterior circulation differences shape the clinical profile of language outcomes.

In summary, focal thalamic strokes are rare both in our cohort (2.5%) and in prior literature (4.9%) (Fritsch et al., 2020) and, when present, were not associated with long-term anomia in our sample. However, we cannot entirely rule out a relationship between focal thalamic lesions and naming impairments due to (a) the small number of patients with focal thalamic damage; (b) the limited extent of thalamic damage (typically partial); and (c) the lack of longitudinal data capturing earlier naming deficits, recovery trajectories, and the transition from acute to chronic stages. Given that statistical power was limited for focal subnuclear analyses, our findings should be interpreted with caution at this finer level of resolution. In addition, although it is possible that anomia is more likely after complete damage to specific thalamic subregions, this is difficult to establish because such lesions rarely occur in stroke, particularly after focal thalamic infarcts that typically result from small perforating arteries and produce partial, patchy damage rather than complete destruction of entire nuclei (Schmahmann, 2003).

### Co-Occurring Thalamic and Non-Thalamic Damage Did Not Worsen Naming

Using whole-brain LSM, we identified two regions where lesion load was significantly associated with naming performance: ROI-TP from LSM-1 and ROI-SC that included the lateral thalamus from LSM-2. Damage to either region increased the likelihood of anomia compared to sparing of both, with the most severe impairments observed when both ROIs were affected.

Although the lateral thalamus was included in the ROI-SC, post hoc analyses found that left lateral thalamic damage did not independently contribute to naming impairments. It was always observed in the context of damage to the caudate, but the combination of damage to both the caudate and the lateral thalamus did not increase the frequency of anomia compared to patients with sparing of both the caudate and the lateral thalamus. In contrast, damage to other parts of the ROI-SC (SLF-III and the insula) was significantly related to naming performance. These findings suggest that the inclusion of the lateral thalamus and caudate in the ROI-SC may be the result of them being incidentally damaged in lesions primarily affecting SLF-III and the insula. Similarly, lesion load in the pulvinar initially appeared to be associated with naming performance independently from other thalamic nuclei, but this relationship disappeared after controlling for damage to the ROI-TP, SLF-III, and the insula. These findings emphasize the critical importance of controlling for co-occurring lesions when evaluating the contribution of small subcortical structures such as the thalamus. Without such controls, apparent associations may reflect collateral damage to adjacent cortical or white matter regions with stronger causal links to behavior.

### Why Might Thalamic Contributions to Naming Not Have Been Detectable Here?

We now generate hypotheses as to why we found no evidence that thalamic damage limits neuroplasticity, disrupts interregional coordination, or impairs executive functions needed for naming. One possibility is that the thalamus does not play a necessary role in long-term naming outcomes and that its role, if any, is supportive or modulatory. Alternatively, the thalamus may contribute to the same naming network as the ROI-TP. Thus, damage to the ROI-TP alone may already produce near-maximal disruption, and additional thalamic involvement yields no measurable additive effect. A third possibility is that multiple (degenerate) naming networks exist: Naming is preserved as long as one system—thalamic or cortical—is intact, and persistent anomia may only emerge when all potential naming networks are compromised. Future studies are therefore required to determine whether fMRI activation in the undamaged left thalamus correlates positively with improvements in naming performance post-stroke and whether this is lesion site dependent.

Finally, a more nuanced and likely possibility is that thalamic contributions to naming are context dependent rather than universally essential and may only emerge under specific conditions or in certain individuals. For example, if the thalamus facilitates naming by supporting attention, working memory, or inter-network coordination (Godefroy et al., 2023; Stockert et al., 2023), then its role may become apparent only under cognitive load (e.g., fatigue, distraction). Such effects are unlikely to be detected unless naming is assessed in variable and demanding contexts such as naming under time pressure. Naming in a nondominant language is a particularly good example of a demanding context, and here, it is relevant to note that basal ganglia–thalamic–cortical activity has been reported to be greater when using the nondominant language compared with the dominant one during a sentence completion task (Quiñones et al., 2024). Future studies could test whether the degree of thalamic involvement is inversely related to proficiency in the nondominant language.

Additionally, in contrast with prior reports, we did not observe long-term fluency impairments following isolated focal thalamic lesions, whereas Rangus et al. (2022, 2024) reported that fluency impairments were prevalent after acute thalamic lesions. Several factors might contribute to this difference, such as cohort characteristics (e.g., acute vs. chronic phase, comorbid non-thalamic damage) and the selection of fluency measures. In the CAT, spoken picture description and timed word-generation task scores assess prompted and executive-driven speech output, indexing production demands that may differ from those captured in clinical rating scales such as the specialized fluency index in the Western Aphasia Battery and therapists’ ratings of spontaneous fluency (in the German Aphasia Checklist). Regardless of these differences, our findings on fluency impairments converge with the naming analyses in showing that long-term deficits were most common when thalamic and non-thalamic regions were jointly damaged.

### Conclusions

Although we did not detect thalamic effects under the conditions of this study, our findings provide meaningful constraints on theories of dominant hemisphere thalamic involvement in language and guide future studies for better understanding thalamic function. First, they challenge the view that the thalamus serves as an irreplaceable node in the naming network. Second, they do not support the hypothesis that the thalamus plays a key role in recovery, since naming impairments were not magnified when thalamic lesions co-occurred with cortical damage. Third, the findings are more compatible with models in which thalamic contributions are distributed, nonobligatory, and variable across individuals. Finally, from a clinical perspective, thalamic lesion location should not be used as an independent predictor of long-term naming outcomes, limiting its utility as a prognostic or diagnostic marker in post-stroke aphasia.

Future research could strengthen and extend these conclusions by (a) using longitudinal designs to bridge acute and chronic stages and track thalamic contributions across recovery stages, (b) studying cases with larger and more focal thalamic lesions to minimize the influence of redundancy on recovery, (c) systematically controlling for co-occurring damage in naming-related regions, (d) using fMRI or magnetoencephalography to assess functional network reorganization after thalamic damage, and (e) adopting high-demand conditions to reveal subtle thalamic effects on naming ability. Additionally, investigating interindividual variability in thalamic activation and connectivity during naming tasks in the neurotypical brain may illuminate the functional heterogeneity and context-dependent dependency of thalamic contributions to language.

## ACKNOWLEDGMENTS

We thank the participants and carers for their generous assistance with our research. We also acknowledge and thank other members of the PLORAS team employed between 2003 and 2020 for their help with the acquisition and organization of behavioral and imaging data.

## FUNDING INFORMATION

Cathy J. Price, Wellcome Trust (https://dx.doi.org/10.13039/100010269), Award ID: 203147/Z/16/Z. Cathy J. Price, Wellcome Trust (https://dx.doi.org/10.13039/100010269), Award ID: 205103/Z/16/Z. Cathy J. Price, Wellcome Trust (https://dx.doi.org/10.13039/100010269), Award ID: 224562/Z/21/Z. Cathy J. Price, Medical Research Council (https://dx.doi.org/10.13039/501100000265), Award ID: MR/M023672/1. Cathy J. Price, Stroke Association (https://dx.doi.org/10.13039/501100000364), Award ID: TSA 2014/02. Alex P. Leff, National Institute for Health and Care Research (https://dx.doi.org/10.13039/501100000272), Award ID: RP-2015-06-012. Jie Zhang, National Natural Science Foundation of China (https://dx.doi.org/10.13039/501100001809), Award ID: 82272592. Jie Zhang, China Scholarship Council (https://dx.doi.org/10.13039/501100004543), Award ID: 202308330272.

## AUTHOR CONTRIBUTIONS

**Jie Zhang**: Formal analysis; Funding acquisition; Visualization; Writing – original draft. **Douglas Neville**: Data curation; Formal analysis; Visualization; Writing – review & editing. **Storm Anderson**: Investigation; Writing – review & editing. **Sophie M. Roberts**: Investigation; Writing – review & editing. **Thomas M. H. Hope**: Methodology; Writing – review & editing. **Alex P. Leff**: Funding acquisition; Writing – review & editing. **David W. Green**: Supervision; Validation; Writing – review & editing. **Cathy J. Price**: Conceptualization; Funding acquisition; Project administration; Supervision; Writing – review & editing.

## CODE AND DATA AVAILABILITY STATEMENTS

The analysis scripts and the accompanying data used in this study are openly accessible via the Open Science Framework at https://osf.io/8ct5p. Raw neuroimaging data contain sensitive information and are therefore not publicly released but can be provided to qualified researchers upon reasonable request from C.J.P. (c.j.price@ucl.ac.uk) and subject to institutional data-sharing agreements.

## TECHNICAL TERMS

Thalamus: A subcortical brain structure that relays sensory and motor signals and supports cognition and consciousness.
Anomia: Word-finding difficulty that occurs during the course of conversational discourse, often assessed clinically with picture-naming tasks.
Aphasia: An acquired communication disorder caused by brain damage, characterized by impairments of language modalities, including speaking, listening, reading, and writing.
Lesion–symptom mapping: A neuroimaging approach linking brain damage locations to specific behavioral impairments.
